# Numerical simulation of the flow of a tangent hyperbolic fluid over a stretching sheet within a porous medium, accounting for slip conditions

**DOI:** 10.1016/j.heliyon.2024.e28683

**Published:** 2024-03-27

**Authors:** Ahmed Alkaoud, Mohamed M. Khader, Ali Eid, Ahmed M. Megahed

**Affiliations:** a Department of Physics, College of Science, Imam Mohammad Ibn Saud Islamic University (IMSIU), Riyadh: 11566, Saudi Arabia; b Department of Mathematics and Statistics, College of Science, Imam Mohammad Ibn Saud Islamic University (IMSIU), Riyadh: 11566, Saudi Arabia; c Department of Mathematics, Faculty of Science, Benha University, Benha, Egypt; d Department of Astronomy, Faculty of Science, Cairo University, Giza, Egypt

**Keywords:** 41A30, 76F12, 65M60, 65N12, Tangent hyperbolic fluid, Thermal radiation, Porous medium, FDM

## Abstract

This study aims to explore the characteristics of tangent hyperbolic fluid flow along a stretching sheet. The sheet has suction or injection influences and is located inside a porous medium. The research inspects the flow and heat transfer (FHT) properties, taking into account the presence of a velocity slip condition. The flow of non-Newtonian magnetohydrodynamic fluid caused by a porous stretching sheet, taking into account thermal radiation and heat generation, has a wide range of engineering applications. These applications involve chemical reactors, energy distribution, storage of solar energy, and filtration processes. Mathematically, the flow problem is expressed as a collection of nonlinear partial differential equations. To numerically solve the resulting ODEs, the finite difference approach (FDM) is successfully used. Tables and graphs are used to display the various output values related to the hyperbolic tangent fluid. Among the different output values that appear are velocity and temperature. Significant observations from the study indicate that an increase in the power-law index, slip velocity parameter, porosity parameter, and magnetic number results in a decrease in the fluid's velocity and an increase in temperature. The completed comparison analysis shows a sizable degree of agreement with the earlier investigation.

## Nomenclature

a: Constant
B0: The intensity of a magnetic field
cp: The heat capacity at constant pressure
Cfx: Coefficient measuring friction on a surface
f: Non-dimensional stream function
k⁎: Coefficient of absorption in the medium
k: The permeability characteristic of the porous medium
M: Magnetic parameter
n: Index of the power-law
Nux: Nusselt number
Pr: Number associated with Prandtl
qr: The flux of radiant heat
Q: Coefficient for the generation of heat
R: Radiation parameter
*Re*: Reynolds number at a specific location
T: Temperature of the fluid following a hyperbolic tangent distribution
Tw: Temperature of the tangent hyperbolic fluid at the sheet
T∞: Temperature of the hyperbolic tangent fluid at a distance from the sheet
u,v: The components of the velocity in the direction of *x*-axis, & *y*-axis respectively
Uw: Velocity associated with stretching
vw: Suction velocity
We: Weissenberg number
x,y: Cartesian coordinates

**Greek symbols**
σ⁎: Constant of Stefan and Boltzmann
κ: Coefficient of conductivity of heat in a fluid
η: Non-dimensional variable
λ0: Coefficient of slip factor
λ: Slip factor
β: Porous parameter
ρ: Density of the tangent hyperbolic fluid
θ: Non-dimensional temperature
ν: Kinematic viscosity
μ: Viscosity factor
Γ: Material time constant
ε: Thermal conductivity parameter
γ: The temperature-dependent heat generation or absorption
δ: The suction parameter

**Superscripts**
′: Derivation with respect to the non-dimensional parameter *η*
w: State at the sheet
∞: State at the ambient.

## 1 Introduction

Since non-Newtonian fluids (NNFs) are widely used in so many different engineering and manufacturing fields, they play an important role in these fields. In the food sector, NNFs are crucial for attaining the correct textures and viscosities in goods like dressings, sauces, and confections [1]. Further, to achieve the appropriate consistency and application qualities, NNFs are used in the formulation of lotions, creams, and gels in the cosmetics sector. The NNFs are also used in oil and gas extraction procedures, making use of their unusual flow characteristics to enhance drilling and pumping techniques. In the pharmaceutical sector, non-Newtonian fluids are employed to create therapeutic combinations that allow for better gradual absorption. Overall, non-Newtonian fluids' adaptability makes them essential in many engineering and manufacturing processes, providing tailored solutions to satisfy particular application requirements [2].

In the context of chemical engineering systems, the tangent hyperbolic fluid (THF) model is significant as a NNF model. In contrast to other NNFs, it has some advantages. Whipped cream, ketchup, blood, nail polish, and many other materials are examples of common materials that use the THF. A study was done by Ghaffar et al. [3] to determine how the Biot number affected the flow of an incompressible, two-dimensional, HTF that was emitted from a sphere. In a study by Shafiq et al. [4], the influences of heat, mass, and motile microbe transfer rates on the bioconvective flow of hyperbolic tangent nanofluid through an angled magnetic field were investigated. In their research, Salahuddin et al. [5] focused on heat generation while studying the magnetohydrodynamic (MHD) fluid flow of a tangent hyperbolic type. To better understand how an incompressible tangent hyperbolic liquid flows over a stretched surface (SS), Mahanthesh et al. [6] merged their research on convective conditions and heat generation. On the flow of a magnetohydrodynamic non-Newtonian hyperbolic tangent fluid through a stretchy sheet, Zakir and Zaman [7] tested the impact of heat transmission. Recognizing the importance of this fluid type, Shahzad et al. [8] recently published a research work examining the behavior of a two-dimensional tangent hyperbolic nanofluid flow past a continuous surface. In a recent study, Khan et al. [9] looked at the implications of double stratification on the convectively heated flow of tangent hyperbolic fluid while taking into account variables such as chemical reaction and nonlinear thermal radiation. The exploration of the tangent hyperbolic fluid flow model, along with its significant engineering and industrial applications, has served as a motivating factor for numerous researchers ([10], [11], [12]), prompting them to conduct extensive investigations in this domain.

In this work, we will use the implicit finite difference approach as a numerical method to solve the problem under study. This method has been incorporated by many papers to solve a wide range of ODEs. Through much research ([13], [14], [15]), this method is a reliable tool for dealing with a wide range of problem types. Using this method, the differential equations that express the model under study can be transformed into a nonlinear system of algebraic equations, then, Newton iteration method is used to solve this system. Many scientists have noted the ability of the FDM to overcome problems and difficulties that may arise during calculations if other numerical methods are used, such as the finite element method [16]. This technique has been used to obtain numerical solutions to many different problems, including the fractional diffusion equation [17], two-sided space fractional wave equation [18], fractional differential equations arising from optimization problems [19], and physical problem of the unsteady Casson fluid flow with heat flux [20].

All the studies mentioned above unmistakably demonstrate that, despite numerous research endeavors revealing the features of the non-Newtonian tangent hyperbolic fluid model, various physical phenomena, including slip velocity studied concurrently with the heat generation phenomenon, remain unexplored in the context of flow and heat transfer problems. Hence, the novelty and objective of our current work are aimed to fill this existing gap. The literature review also indicates the absence of any previous endeavors to examine the impacts of thermal radiation on MHD slippery THF flow through a Darcy porous medium in the presence of a linear SS. Motivated by these analyses, this research paper undertakes a numerical exploration of the dynamics of a magnetohydrodynamic slip flow of a tangent hyperbolic fluid over an SS embedded in a porous medium with variable thermal conductivity. The governing boundary layer (BL) equations for flow and temperature distribution are transformed and subsequently numerically treated by using the finite difference method. Utilizing plotted graphs, a comprehensive analysis and discussion of the relevant physical parameters are presented, contributing to a detailed comprehension of their behavior.

## 2 Formulation of the problem

**Figure 1:**
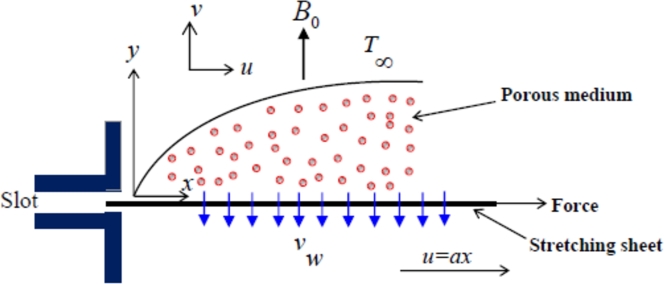
Schematic diagram of the physical model.

This research investigates the characteristics of a continuous, 2-Dim. flow involving an incompressible fluid featuring a tangent hyperbolic profile. The fluid moves adjacent to a stretched surface located at y=0. The study specifically focuses on the region where the value of *y* exceeds 0. This specific fluid displays a unique stress tensor, and extensively explained in [21]. Additionally, we assume that the flow is initiated by the linear elongation of the sheet. Furthermore, the study delves into understanding the traits of fluid FHT within a porous medium characterized by a permeability coefficient identified as *k*. In this study, the magnetic field is taken as constant and consistent, maintaining a steady strength of $B_{0}$ across the analyzed boundary region. The analysis encompasses a velocity component, denoted as *u*, which is directed along the *x*-axis and arises from the stretching process applied to the elastic sheet. The magnetic field, depicted in Fig. 1, is presumed to be aligned perpendicular to the sheet's surface in the *y*-direction. This implies that the orientation of the magnetic field is such that it forms a right angle with the surface of the sheet along the *y*-axis, as illustrated in Fig. 1.

The obvious goal of this study is to investigate the phenomenon of heat generation, characterized by the coefficient *Q*, which occurs throughout the fluid flow process within the analyzed system. The emphasis is placed on comprehending the underlying mechanisms and accurately quantifying the heat transfer processes associated with it. The premise underpinning this phenomenon follows the relationship below [22]:

$$Q=\frac{\kappa U_{w}}{x\nu }\gamma (T-T_{\infty }).$$

It is important to note that the parameter *Q* represents heat generation when Q>0, while Q<0 signifies heat consumption. Similarly, within the system, $T_{\infty }$ is the ambient fluid temperature, $U_{w}$ is the stretching velocity, which can be assumed as $U_{w}=ax$, and *ν* represents the kinematic viscosity. Also, the parameter *γ* represents temperature-dependent heat generation or absorption. When *γ* is positive, it indicates heat generation, whereas a negative value of *γ* signifies heat absorption.

Incorporating a radiative heat flux is a crucial tenet of our physical model. This presumption suggests that radiative heat transfer plays a considerable role in the system's overall heat transfer process. The radiative heat flux $q_{r}$ is a major component of this model and is defined by the following relation [23]:

(1) $$q_{r}=-\frac{4\sigma^{⁎}}{3k^{⁎}}\frac{\partial T^{4}}{\partial y}.$$

$q_{r}$ and its interactions with the system are greatly influenced by these characteristics. It is assumed that the temperature difference $T^{4}$ within the flow is taken as a linear combination of temperatures. We obtain the following formulation by using Taylor's series and only taking into account the low-order terms [24]:

(2) $$T^{4}≅-3T_{\infty }^{4}\left(1-\frac{4T}{3T_{\infty }}\right).$$

The Equation (2) provides a clear means of simplifying Equation (1) into the following form:

$$q_{r}=-\frac{16\sigma^{⁎}T_{\infty }^{3}}{3k^{⁎}}\left(\frac{\partial T}{\partial y}\right).$$

The distributions of velocity and temperature in a two-dimensional space are governed by the equations of momentum and thermal energy under the previous assumptions. These equations can be visualized using the methods described by Ullah et al. [25]:

(3) $$\frac{\partial u}{\partial x}+\frac{\partial v}{\partial y}=0,$$

(4) $$u\frac{\partial u}{\partial x}+v\frac{\partial u}{\partial y}=(1-n)\nu \frac{\partial^{2}u}{\partial y^{2}}+\sqrt{2}\;\nu \;n\Gamma \;\frac{\partial u}{\partial y}\frac{\partial^{2}u}{\partial y^{2}}-\frac{\mu }{\rho k}u-\frac{\sigma B_{0}^{2}}{\rho }u,$$

(5) $$u\frac{\partial T}{\partial x}+v\frac{\partial T}{\partial y}=\frac{1}{\rho c_{p}}\frac{\partial }{\partial y}(\kappa (T)\frac{\partial T}{\partial y})+\frac{Q}{\rho c_{p}}-\frac{1}{\rho c_{p}}\frac{\partial q_{r}}{\partial y}.$$

The parameters $\rho ,\Gamma ,c_{p},n$, and *σ* represent the density, material time constant, specific heat at constant pressure, power law index, and electrical conductivity, respectively.

Now, it is possible to represent the corresponding conditions at the model's boundaries through written statements:

$$v=-v_{w},\;\;\;\;\;u=U_{w}+\lambda_{0}\left((1-n)\frac{\partial u}{\partial y}+\frac{n\Gamma }{\sqrt{2}}{\left(\frac{\partial u}{\partial y}\right)}^{2}\right),\;\;\;\;\;T=T_{w},\;\;\;\;\;\text{at}\;\;\;\;\;y=0,$$

$$u\to 0,\;\;T\to T_{\infty },\;\;\text{as},\;\;y\to \infty .$$

In simple terms, $v_{w}$ denotes the speed at which suction or injection occurs. $\lambda_{0}$ represents the coefficient that measures the difference in velocity between the sheet surface and the moving fluid. Finally, $T_{w}$ stands for the temperature of the sheet being referred to.

Now, the first step in using the proposed numerical technique for a solution is to transform the partial differential equations from Equations (3)-(5) into ordinary differential equations. The method outlined in reference [24] can be used to accomplish this transition as follows:

$$\eta =y\sqrt{a\;\rho \mu^{-1}},\;\;\theta (\eta )=(T-T_{\infty }){\left(T_{w}-T_{\infty }\right)}^{-1},\;\;u=axf^{\prime }(\eta ),\;\;v=-\sqrt{a\mu \rho^{-1}}f(\eta ).$$

After applying these transformations, the resulting differential equations describing the reduced flow can be defined in the following forms:

(6) $$\left[(1-n)+n\;W_{e}\;f^{\prime \prime }\right]f^{\prime \prime \prime }+ff^{\prime \prime }-f^{\prime 2}-\left(M+\beta \right)f^{\prime }=0,$$

(7) $$\frac{1}{\mathrm{Pr}}\left[(1+R+\varepsilon \;\theta )\theta^{\prime \prime }+\varepsilon \;\theta^{\prime \;2}\right]+f\theta^{\prime }+\frac{\gamma }{\mathrm{Pr}}\left(1+\varepsilon \;\theta \right)\theta =0,$$

(8) $$f(0)=\delta ,\;\;\;f^{\prime }(0)=1+\lambda \left[(1-n)f^{\prime \prime }(0)+\frac{nW_{e}}{2}f^{\prime \prime 2}(0)\right],\;\;\;\theta (0)=1,$$

(9) $$f^{\prime }\to 0,\;\;\theta \to 0,\;\;\text{as},\;\eta \to \infty .$$

All parameters (the suction parameter, magnetic parameter, Weissenberg number, porous parameter, slip velocity parameter, Prandtl number, and radiation parameter) relied upon by the previous system can be interpreted or defined respectively as follows:

$$\delta =\frac{v_{w}}{\sqrt{a\nu }},\;\;\;M=\frac{\sigma B_{0}^{2}}{\rho \;a},\;\;\;W_{e}=\frac{\sqrt{2}a^{\frac{3}{2}}x\Gamma }{\sqrt{\nu }},\;\;\;\beta =\frac{\mu }{ak\rho },$$

$$\lambda =\lambda_{0}\sqrt{\frac{a}{\nu }},\;\;\;\mathrm{Pr}=\frac{\mu \;c_{p}}{\kappa_{\infty }},\;\;\;R=\frac{16\sigma^{⁎}T_{\infty }^{3}}{3\kappa_{\infty }k^{⁎}}.$$

## 3 Engineering and industrial quantities

Two significant physical quantities have a significant impact on engineering and practical applications. It is crucial to comprehend and evaluate each of these amounts separately. These quantities are the local Nusselt number $Nu_{x}$ (LNN), and the local skin-friction coefficient $Cf_{x}$ (LSFC) and provided below:

$$Cf_{x}Re^{\frac{1}{2}}=-\left((1-n)f^{\prime \prime }(0)+\frac{nW_{e}}{2}f^{\prime \prime 2}(0)\right),\;\;\;Nu_{x}Re^{\frac{-1}{2}}=-\theta^{\prime }(0),$$

where $Re=\frac{U_{w}x}{\nu }$ is the local Reynolds number.

## 4 Numerical solutions by implementing FDM

This section is devoted to applying the FDM to provide numerical solutions to Equations (6)-(7), which represent the proposed system under study with the boundary conditions (8)-(9). Previously, through many published works, this method has been tested for its accuracy and efficiency in solving different problems. To enable the use of this method, we will use the transformation $f^{\prime }(\eta )=\phi (\eta )$ to rewrite the system of Equations (6)-(7) as follows:

(10) $$f^{\prime }-\phi =0,$$

(11) $$\left[(1-n)+n\;W_{e}\;\phi^{\prime }\;\right]\phi^{\prime \prime }+f\;\phi^{\prime }-\phi^{2}-\left(M+\beta \right)\;\phi =0,$$

(12) $$\left[(1+R+\varepsilon \;\theta )\theta^{\prime \prime }+\varepsilon \;\theta^{\prime \;2}\right]+\mathrm{Pr}\;\;f\theta^{\prime }+\gamma \;\left(1+\varepsilon \;\theta \right)\theta =0,$$

(13) $$f(0)=\delta ,\;\;\;\phi (0)=1+\lambda \left[(1-n)\;\phi^{\prime }(0)+0.5\;n\;W_{e}\;\phi^{\prime 2}(0)\right],\;\;\;\theta (0)=1,$$

(14) $$\phi (\eta_{\infty })=0,\;\;\;\theta (\eta_{\infty })=0.$$

In the FDM, the domain of the problem is divided into some discrete subintervals across a set of nodes. For this, we use the symbols; Δη=ħ>0 to be the mesh size in the *η*-direction, $\Delta \eta =\eta_{\infty }/N$, with $\eta_{j}=j\;\hbar$ for j=0,1,...,N. Define $f_{j}=f(\eta_{j}),\;\;\phi_{j}=\phi (\eta_{j})$, and $\theta_{j}=\theta (\eta_{j})$.

Let f,ϕ, and *θ* approximate by the numerical values $F_{j}$, $\Phi_{j}$, and $\Theta_{j}$, respectively at the $j^{th}$ node. We define

(15) $$f^{\prime }{|}_{j}\approx \frac{f_{j+1}-f_{j-1}}{2\hbar },\;\;\;\phi^{\prime }{|}_{j}\approx \frac{\phi_{j+1}-\phi_{j-1}}{2\hbar },\;\;\;\theta^{\prime }{|}_{j}\approx \frac{\theta_{j+1}-\theta_{j-1}}{2\hbar },$$

(16) $$\phi^{\prime \prime }{|}_{j}\approx \frac{\phi_{j+1}-2\phi_{j}+\phi_{j-1}}{\hbar^{2}},\;\;\;\theta^{\prime \prime }{|}_{j}\approx \frac{\theta_{j+1}-2\theta_{j}+\theta_{j-1}}{\hbar^{2}}.$$

One of the basics of applying the FDM is to express the system being solved in a discretized form, then after that we replace from (15)-(16) into the model (10)-(14). By neglecting truncation errors, this system of ODEs turns into the following system of non-linear algebraic equations:

(17) $$F_{k+1}-F_{k-1}-2\hbar \;\Phi_{k}=0,\;\;\;k=0,1,...,N,$$

(18) $$[2\hbar^{2}\;(1-n)+\hbar \;n\;W_{e}\;(\Phi_{k+1}-\Phi_{k-1})](\Phi_{k+1}-2\Phi_{k}+\Phi_{k-1})+\hbar \;F_{k}\;(\Phi_{k+1}-\Phi_{k-1})-2\hbar^{2}\;\Phi_{k}\left(\Phi_{k}+M+\beta \right)=0,$$

(19) $$4(1+R+\varepsilon \;\Theta_{k})(\Theta_{k+1}-2\Theta_{k}+\Theta_{k-1})+\varepsilon \;{\left(\Theta_{k+1}-\Theta_{k-1}\right)}^{2}+2\hbar \;\mathrm{Pr}\;\;F_{k}(\Theta_{k+1}-\Theta_{k-1})+4\gamma \;\hbar^{2}\;\left(1+\varepsilon \;\Theta_{k}\right)\Theta_{k}=0.$$

Also, the boundary conditions are:

(20) $$\Phi_{0}=1+\lambda \left[(1-n)\;\hbar^{-1}\;(\Phi_{1}-\Phi_{0})+0.5\;n\;W_{e}\;\hbar^{-2}\;{\left(\Phi_{1}-\Phi_{0}\right)}^{2}\right],F_{0}=\delta ,\;\;\;\;\Theta_{0}=1,\;\;\;\;\Phi_{N}=0,\;\;\;\;\Theta_{N}=0.$$

Now we use Newton iteration method as one of the most important and efficient numerical methods in solving systems of nonlinear algebraic equations (17)-(20) to obtain the approximations $F_{j},\;\;\Phi_{j}$, and $\Theta_{j}$, (j=0,1,...,N). In our calculation, we used a suitable initial solution to solve this system with the help of the Mathematica Package, and Newton iteration method.

## 5 Validation of the code's accuracy

A thorough analysis is shown in Table 1 to confirm the accuracy of the suggested FDM, with the mesh size ħ=0.25. The table gives a summary of the outcomes from applying the approach to different Weissenberg values $W_{e}$, concentrating on the skin-friction coefficient. These conclusions were drawn from a prior issue that Ullah et al. [25] looked into. The comparison demonstrates the method's capacity to faithfully recreate well-known solutions and precisely forecast physically relevant quantities. These findings in Table 1 provide persuasive evidence for the validity and dependability of the suggested numerical method, confirming its suitability as a reliable and accurate instrument for the intended physical problem.

**Table 1:** An examination of skin friction coefficients $Cf_{x}Re^{\frac{1}{2}}$ in relation to previous research of Ullah et al. [25] for assorted quantities of *W*_*e*_ when *β* = *λ* = *M* = 0 and *n* = 0.1.

*W*_*e*_	Ullah et al. [25]	Current work
0.0	0.94967	0.949666301
0.3	0.94345	0.943447810
0.5	0.93922	0.939218895

## 6 Interpretation of numerical results

**Figure 2:**
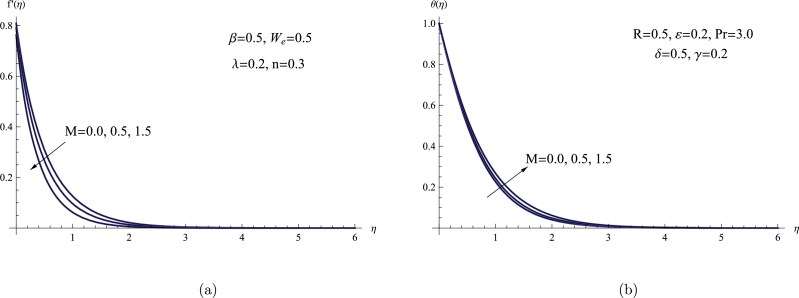
(a) *f*′(*η*) for various *M*, (b) *θ*(*η*) for various *M*.

Equations (6) and (7) of a system of coupled nonlinear ODEs were computed numerically using the FDM in Mathematica software version 11. During the process of finding a solution, the boundary conditions (8) and (9) were also taken into consideration. This section focuses on analyzing the impact of different physical parameters, including *M*, *β*, $W_{e}$, Pr, *λ*, *n*, *δ*, and *R* on various flow characteristics. These characteristics include the LNN, dimensionless velocity profiles $f^{\prime }(\eta )$, LSFC, and temperature profiles θ(η). The effects of the magnetic number *M* on $f^{\prime }(\eta )$ and θ(η) are shown in Fig. 2(a,b). The findings shown in Fig. 2 unmistakably illustrate that when *M* rises, the $f^{\prime }(\eta )$ noticeably declines as θ(η) rises. Additionally, it has been found that lower magnetic numbers result in a thicker BL. In a physical sense, the elevation of the magnetic parameter enhances the interplay between the magnetic field and the fluid, causing alterations in the thermal characteristics of the system. Consequently, this leads to an increased temperature distribution within the fluid. Moreover, the influence of *M* on the velocity characteristics of fluid flow can be observed similarly to what was presented in prior publications [26], thereby validating our findings from a physical perspective.

**Figure 3:**
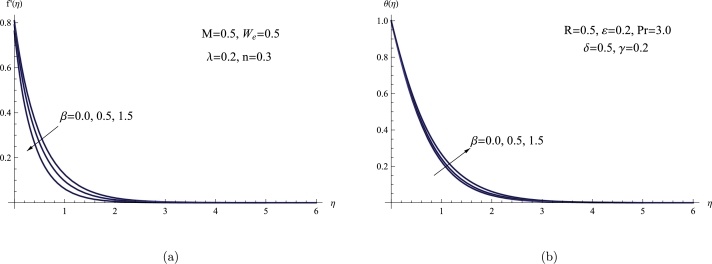
(a) *f*′(*η*) for various *β*, (b) *θ*(*η*) for various *β*.

The consequence of *β* on the profiles of θ(η) and $f^{\prime }(\eta )$ is depicted graphically in Fig. 3(a,b). The plot clearly shows that the BL thickness and the magnitude of the velocity simultaneously drop as the porosity parameter grows. The θ(η) exhibits the opposite behavior with the same parameter, exhibiting an increase, in contrast to previous tendencies. The presence of *β* serves as a fundamental physical rationale for the reduction in velocity associated with an increase in *β*. With a rise in the porosity parameter, the porous medium becomes more adept at impeding fluid flow. The porous material acts as a hindrance, establishing a barrier that intensifies resistance and hinders the smooth movement of the fluid. Consequently, as the fluid encounters heightened resistance within the porous material, its velocity undergoes a deceleration.

**Figure 4:**
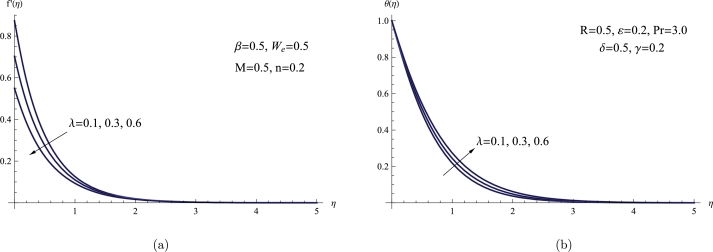
(a) *f*′(*η*) for various *λ*, (b) *θ*(*η*) for various *λ*.

Fig. 4(a,b) demonstrates how $f^{\prime }(\eta )$ and θ(η) profiles are affected by variations in the velocity slip parameter *λ*. According to the plot, the sheet velocity θ(0), the BL thickness, and the magnitude of the velocity field all diminish as the value of *λ* rises. On the other hand, the thermal thickness and temperature distribution exhibit a different pattern, increasing with higher values of *λ*. Physically, a heightened slip effect, denoted by an escalation in the slip velocity parameter *λ*, signifies a more pronounced phenomenon wherein fluid molecules close to the surface experience diminished contact. This decreased interaction imposes constraints on the transfer of momentum between the fluid and the surface, consequently resulting in a decline in velocity.

**Figure 5:**
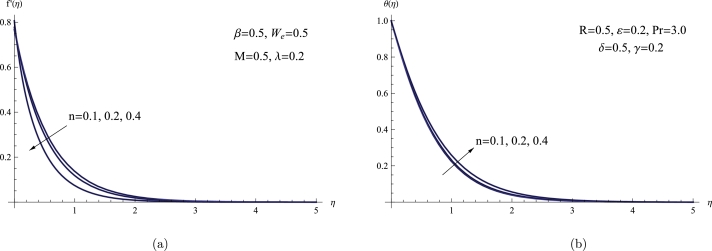
(a) *f*′(*η*) for various *n*, (b) *θ*(*η*) for various *n*.

Fig. 5(a,b) shows how *n* affects on θ(η) and $f^{\prime }(\eta )$ profiles. It is obvious that when *n* rises, θ(η) rises while $f^{\prime }(\eta )$ falls. From a physical perspective, an increase in viscosity is produced when *n* rises. The result is a drop in fluid velocity as a result of an upsurge in viscosity. Physically, the decline in velocity distribution associated with an elevated power law index parameter is attributed to its impact on the rheological properties of the fluid. A higher power law index parameter signifies a departure from Newtonian fluid behavior, resulting in heightened viscosity and resistance within the fluid.

**Figure 6:**
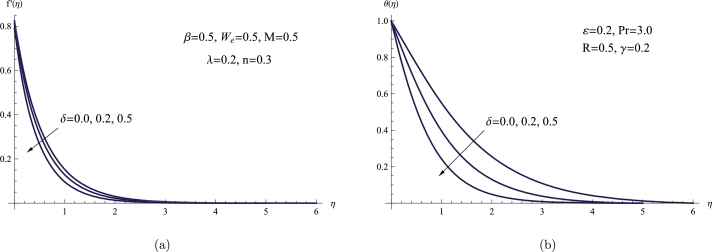
(a) *f*′(*η*) for various *δ*, (b) *θ*(*η*) for various *δ*.

To analyze θ(η) and $f^{\prime }(\eta )$ while taking the suction parameter *δ* into consideration, Fig. 6(a,b) is built in this manner while maintaining the constant values of the other parameters. The strength or intensity of the boundary layer thickness, temperature distribution, and velocity distribution all decrease with increasing the suction parameter. Physically, the strength of the suction imparted to the fluid is represented by the suction parameter, which causes the velocity distribution to drop as it increases. In addition, the fluid can't sustain higher velocities when the suction parameter increases because it feels a stronger pulling force. Because of the resistance created by this higher suction, the fluid's velocity dispersion decreases.

**Figure 7:**
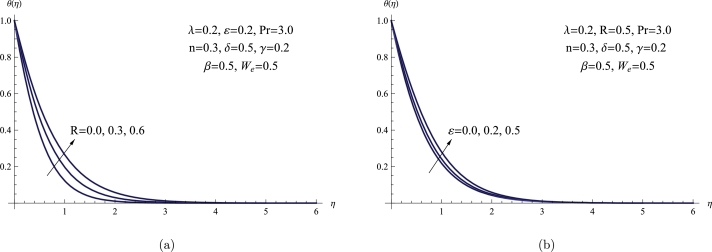
(a) *θ*(*η*) for various *R*, (b) *θ*(*η*) for various *ε*.

Fig. 7(a,b) shows the changes in fluid temperature θ(η) resulting from the influence of *ε* and *R* parameters. When *ε* and *R* increase, the temperature of the non-Newtonian fluid also increases. However, in scenarios where there is no thermal radiation and the thermal conductivity is low, the tangent hyperbolic fluid demonstrates a comparatively lower temperature. Mathematically, *ε* is directly proportional to the ambient fluid temperature. Consequently, a higher radiation parameter is obtained from the higher ambient fluid temperature, leading to an increase in both the temperature distribution and the thermal thickness.

**Figure 8:**
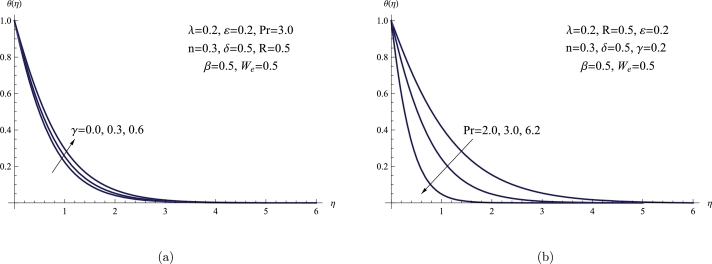
(a) *θ*(*η*) for various *γ*, (b) *θ*(*η*) for various *Pr*.

Fig. 8(a,b) elucidates the variations in θ(η) in response to *γ* and *Pr*. According to the plot, as the heat generation parameter elevates, θ(η) and the related BL thickness also rise. The θ(η) and BL thickness, on the other hand, fall as *Pr* rises, which is an entirely different response. Physically, when the fluid thermal conductivity coefficient falls, the normal response is for *Pr* to rise, which lowers θ(η). Furthermore, the effect of heat generation on the temperature traits of fluid flow can be observed in a manner consistent with what was previously reported in publications [27], thus confirming the validity of our findings from a physical standpoint.

In Table 2, a tabular representation of the effects of various governing parameters on the LNN and LSFC is shown. The table clearly shows that the LNN and LSFC are affected in the same ways by *M* and *β*. It is clear that both values lower the LNN and raise the LSFC. Heat transport phenomena are greatly influenced by the existence of thermal radiation, heat generation, and thermal conductivity characteristics. It has been noted that the Nusselt number falls when these parameters rise. As *n* and *λ* rise, it is shown that the LNN and the LSFC also drop. Additionally, the LNN and the LSFC exhibit an increasing trend concerning the suction parameter.

**Table 2:** Values of $Re_{x}^{\frac{-1}{2}}Nu_{x}$ and $Re_{x}^{\frac{1}{2}}Cf_{x}$ for different values of *M*,*β*,*λ*,*n*,*δ*, *R*,*ε*,*γ* and *Pr* with *W*_*e*_ = 0.5.

*M*	*β*	*λ*	*n*	*δ*	*R*	*ε*	*γ*	*Pr*	$Re_{x}^{\frac{1}{2}}Cf_{x}$	$Re_{x}^{\frac{-1}{2}}Nu_{x}$
0.0	0.5	0.2	0.3	0.5	0.5	0.2	0.2	3.0	0.957859	1.22571
0.5	0.5	0.2	0.3	0.5	0.5	0.2	0.2	3.0	1.048420	1.18857
1.5	0.5	0.2	0.3	0.5	0.5	0.2	0.2	3.0	1.188941	1.13277

0.5	0.0	0.2	0.3	0.5	0.5	0.2	0.2	3.0	0.957859	1.22571
0.5	0.5	0.2	0.3	0.5	0.5	0.2	0.2	3.0	1.048420	1.18857
0.5	1.5	0.2	0.3	0.5	0.5	0.2	0.2	3.0	1.188941	1.13277

0.5	0.5	0.1	0.3	0.5	0.5	0.2	0.2	3.0	1.270401	1.25127
0.5	0.5	0.3	0.3	0.5	0.5	0.2	0.2	3.0	0.991831	1.17835
0.5	0.5	0.6	0.3	0.5	0.5	0.2	0.2	3.0	0.751591	1.10514

0.5	0.5	0.2	0.1	0.5	0.5	0.2	0.2	3.0	1.166710	1.22759
0.5	0.5	0.2	0.2	0.5	0.5	0.2	0.2	3.0	1.115231	1.21224
0.5	0.5	0.2	0.4	0.5	0.5	0.2	0.2	3.0	0.968417	1.15488

0.5	0.5	0.2	0.3	0.0	0.5	0.2	0.2	3.0	0.879612	0.45633
0.5	0.5	0.2	0.3	0.2	0.5	0.2	0.2	3.0	0.944465	0.74384
0.5	0.5	0.2	0.3	0.5	0.5	0.2	0.2	3.0	1.048421	1.18857

0.5	0.5	0.2	0.3	0.5	0.0	0.2	0.2	3.0	1.048421	1.64203
0.5	0.5	0.2	0.3	0.5	0.3	0.2	0.2	3.0	1.048421	1.33637
0.5	0.5	0.2	0.3	0.5	0.6	0.2	0.2	3.0	1.048421	1.12621

0.5	0.5	0.2	0.3	0.5	0.5	0.0	0.2	3.0	1.048421	1.34616
0.5	0.5	0.2	0.3	0.5	0.5	0.2	0.2	3.0	1.048421	1.18857
0.5	0.5	0.2	0.3	0.5	0.5	0.5	0.2	3.0	1.048421	1.00697

0.5	0.5	0.2	0.3	0.5	0.5	0.2	0.0	3.0	1.048421	1.27008
0.5	0.5	0.2	0.3	0.5	0.5	0.2	0.3	3.0	1.048421	1.14421
0.5	0.5	0.2	0.3	0.5	0.5	0.2	0.6	3.0	1.048421	0.99110

0.5	0.5	0.2	0.3	0.5	0.5	0.2	0.2	2.0	1.048421	0.75849
0.5	0.5	0.2	0.3	0.5	0.5	0.2	0.2	3.0	1.048421	1.18857
0.5	0.5	0.2	0.3	0.5	0.5	0.2	0.2	6.2	1.048421	2.36481

Remark Just as we mentioned earlier, the FDM is one of the numerical methods with more accuracy and high efficiency. It is also known that the error in each of the approximations of the first and second derivatives, which are defined in equations (15)-(16) is of the order o($\hbar^{2}$). Therefore, we find that the convergence and stability of the used numerical scheme depend greatly on the size of the mesh step *ħ*, i.e., if the value of *ħ* decreases, then the value of the error decreases. So *ħ* is the parameter that controls the stability of the resulting solutions. This is clear in light of the tabular or graphical results, as well as the comparisons made with a special case of the model under study and using another numerical method (see Table 1).

## 7 Conclusions

This study evaluated the outcomes of a theoretical investigation on the impact of slip velocity, thermal radiation, and heat generation on the movement of tangent hyperbolic fluid over the SS. The evaluation was carried out using the FDM. The heat transfer mechanism was accurately described by applying the variable thermal conductivity of the fluid. Additionally, a limitation was imposed on the permeability of the sheet through the implementation of a suction velocity. To verify the accuracy and reliability of the proposed numerical technique, validation for the problem is conducted to ensure its authentication. The given approximate solutions by applying the suggested FDM closely align with the physical interpretation of the problem, indicating the effectiveness and accuracy of the proposed approach. This successful outcome validates the method and highlights its significant potential. The study mentioned above yields the following key findings:

1. As the suction parameter values rise, there is a decrease observed in both the energy and velocity distributions.
2. The decline in the rate of heat transfer gets more pronounced, especially when the parameters M,n,k, and *λ* all improve.
3. During the heat transfer process, the radiation parameter, thermal conductivity parameter, and heat generation parameter play crucial roles in determining the thermal performance.
4. Enhancing values of *λ* and *n* parameters lead to a drop in velocity profiles, while simultaneously intensifying the thermal field.
5. The skin-friction coefficient is influenced in opposite ways by the slip velocity and power-law index parameters.

**Consent for publication**

All authors on this paper agreed to publish.

## CRediT authorship contribution statement

**Ahmed Alkaoud:** Writing – review & editing, Supervision, Methodology, Investigation, Formal analysis. **Mohamed M. Khader:** Writing – original draft, Validation, Software, Methodology, Formal analysis. **Ali Eid:** Writing – original draft, Visualization, Validation, Resources, Investigation, Formal analysis. **Ahmed M. Megahed:** Writing – original draft, Software, Resources, Investigation, Formal analysis, Conceptualization.

## Declaration of Competing Interest

The authors declare that they have no known competing financial interests or personal relationships that could have appeared to influence the work reported in this paper.

## Data Availability

The data of this study will be available from the corresponding author on reasonable request.
